# 

**Published:** 1887-03

**Authors:** 


WHOLE NUMBER CCCII. MARCH, 1887. NUMBER 8. VOLUME XXVI.

INDEX MEDICUS
LIBRARY INDEX

### THE BUFFALO Medical AND Surgical Journal

ESTABLISHED IN YEAR 1844.

**EDITORS:**

**THOS. LOTHROP, M. D.** **A. R. DAVIDSON, M. D.**

ASSOCIATE EDITORS:

FLOYD S. CREGO, M. D. CARLTON C. FREDERICK, M. D.
FRED. R. CAMPBELL, M. D. HERBERT MICKLE, M. D.

EDWARD B. ANGELL, M. D., Rochester, N. Y.

